# miR-22-3p is involved in gluconeogenic pathway modulated by 3,5-diiodo-L-thyronine (T2)

**DOI:** 10.1038/s41598-019-53019-2

**Published:** 2019-11-12

**Authors:** Rosalba Senese, Federica Cioffi, Giuseppe Petito, Pieter de Lange, Aniello Russo, Fernando Goglia, Antonia Lanni, Nicoletta Potenza

**Affiliations:** 1 Department of Environmental, Biological and Pharmaceutical Sciences and Technologies, University of Campania “L. Vanvitelli”, Caserta, Italy; 2 Department of Sciences and Technologies, University of Sannio, Benevento, Italy

**Keywords:** Extracellular signalling molecules, Pre-diabetes

## Abstract

The 3,5-diiodo-L-thyronine (T2) has emerged as an active iodothyronine and its beneficial effects on glucose metabolism including glucose tolerance and insulin resistance is well established. However, little is known about its molecular mechanisms. Given the emerging importance of microRNAs in various metabolic diseases, in this study a possible link between the effects of T2 on glucose metabolism and miRNA expression was investigated by using an *in vivo* model in which T2 was administered in rats receiving a high fat diet, a condition known to impair glucose homeostasis. The results showed that T2-treated rats had a better tolerance to glucose load and a better performance at the insulin tolerance test in comparison to high fat diet animals. Interestingly, in the serum of the animals treated with T2 there was a general decrease of miRNAs with miR-22a-3p, miR-34c-5p and miR-33a-3p significantly downregulated. Furthermore, miR-22a-3p had the largest variation pointing toward its preeminent role in T2 metabolic effect. In fact, in liver there was an up-regulation of its target (Transcription Factor 7) Tcf7, which had an important impact on gluconeogenesis. This study provide, for the first time, evidences that miRNAs are involved in the effects exerted by T2 on glucose homeostasis.

## Introduction

Thyroid hormones (THs; 3,5,3′,5′-tetraiodo-L-thyronine,T4 and 3,5,3′-triiodothyronine, T3) are essential for many physiological processes such as growth, differentiation and metabolism^1,2^. In the last years, several data have shown that some THs derivatives (natural metabolites and analogues) play a relevant biological role on the metabolism (for reviews see^3–8^). Among these, many studies are focused on 3,5-diiodo-L-thyronine (T2), a biologically active iodothyronine^9–11^ (for review see^7,12–15^). In rats fed on a high-fat diet (HFD), the simultaneous administration of T2 (25 μg/100 g body weight for 4 weeks) prevented hyperlipidemia, liver adiposity, body weight gain and insulin resistance^16–18^ and, at that dose, no signs of thyrotoxicosis were detected^16,17^. Interestingly, it has been also shown that T2 administration to HFD-fed rats improved glucose tolerance, insulin sensitivity^18^ and gluconeogenesis in hepatocytes^17^. Furthermore, a possible physiological link between glucose metabolism^19,20^ and circulating T2 has been highlighted by findings from a healthy euthyroid population. To date, however, the mechanism by which T2 affects glucose homeostasis remains to be clarified.

Many reports have demonstrated the involvement of miRNAs in various diseases^21–23^, mainly in the etiology of cancer but also in metabolic disorders such as obesity and type 2 diabetes (T2D)^24,25^ which, in turn, are characterized by an impaired glucose homeostasis^26,27^. Indeed, these small non-coding RNA molecules modulate the levels of kinases and enzymes in the glucose metabolism^28^ by affecting posttranscriptional regulation of gene expression. Particularly, an altered hepatic miRNA signature for obese diabetic *db*/*db* mice was identified by comprehensive miRNome and in silico analyses^29^. This report showed an altered Wnt signaling pathways in diabetic liver^29^, and various lines of evidence suggest that Wnt/β-catenin signaling regulates hepatic glucose production^28,30–32^.

Based on those considerations, the aim of this study was to examine the potential involvement of miRNAs in the mechanisms of action by which T2 affects glucose homeostasis in rats fed on a high-fat-diet. This animal model was chosen since HFD treated rats showed an altered glucose homeostasis and some metabolic disorders, such as altered glucose tolerance and insulin-resistance, thus revealing the beneficial effect of a T2 treatment. In particular: a) a screening of circulating miRNA changes in the serum from HFD-T2 treated vs HFD rats was performed to identify miRNAs potentially involved in the effects of T2; b) the top-ranked microRNA from the screening, rno-miR-22-3p (miR-22), was further investigated in relation to its possible transcript targets involved in glucose metabolism.

## Results

### Characterization of animal models

Three experimental rat groups were analyzed in this study: rats receiving a standard diet (N), rats receiving a high-fat diet (HFD) and rats receiving a high-fat diet (HFD) with simultaneous administration of T2 (HFD-T2). The metabolic parameters of the three experimental groups were fully determined (Table 1). After four weeks of treatment, HFD-T2 rats had accumulated less fat. In fact, they were 14% lighter compared to the HFD animals (Table 1) with a body weight not different from that of N animals, while the HFD rats were overweight, weighing ~14% more compared to N rats (Table 1). Furthermore, in HFD-T2 rats there were a significantly decrease in adipose mass (Table 1) responsible, primarily, for the lower body weight observed (Table 1). The percentage ratio visceral fat pad weight/body weight was 3.2 ± 0.32 in HFD rats against 2.75 ± 0.153 in HFD-T2 ones (P < 0.05; Table 1). These results are consistent with the action of T2 in counteracting gain in body weight and fat accumulation during an HFD diet^33,34^. Heart and skeletal muscle weight, important thyrotoxicosis markers, did not differ among the groups (Table 1). Moreover, in HFD-T2 animals serum levels of cholesterol and triglycerides were comparable to those of N rats while were significantly higher in the HFD group (Table 1).

**Table 1:** T2 administration prevents HFD-induced changes in systemic metabolic parameters and fat accumulation.

	N	HFD	HFD-T2
BW (g)	405 ± 10	470 ± 12.6*	410 ± 13.2^#^
LW/BW (%)	2.7 ± 0.23	3.18 ± 0.18	2.71 ± 0.22
HW/BW (%)	0.3 ± 0.01	0.25 ± 0.01	0.309 ± 0.02
GW/BW	0.6 ± 0.04	0.5 ± 0.02	0.55 ± 0.03
WW/BW	2.8 ± 0.2	3.2 ± 0.32*	2.75 ± 0.15^#^
Cholesterol mg/dl	55 ± 2.5	80 ± 3.7*	60 ± 2.36^#^
Triglycerides	125 ± 8.12	250 ± 10.2*	124 ± 10.5^#^

Body Weight (BW), percentage ratio liver weight/body weight (LW/BW); percentage ratio heart weight/body weight (HW/BW); percentage ratio gastrocnemius weight/body weight (GW/BW); percentage ratio white adipose tissue weight/body weight (WW/BW); and serum cholesterol levels in rats fed on to standard diet (N), to high-fat diet (HFD) and to HFD with contemporary administration of T2 (HFD-T2).*p < 0.05vs N; ^#^P < 0.05 vs HFD. Values are mean ± SEM of 5 independent treatments.

**Figure 1:**
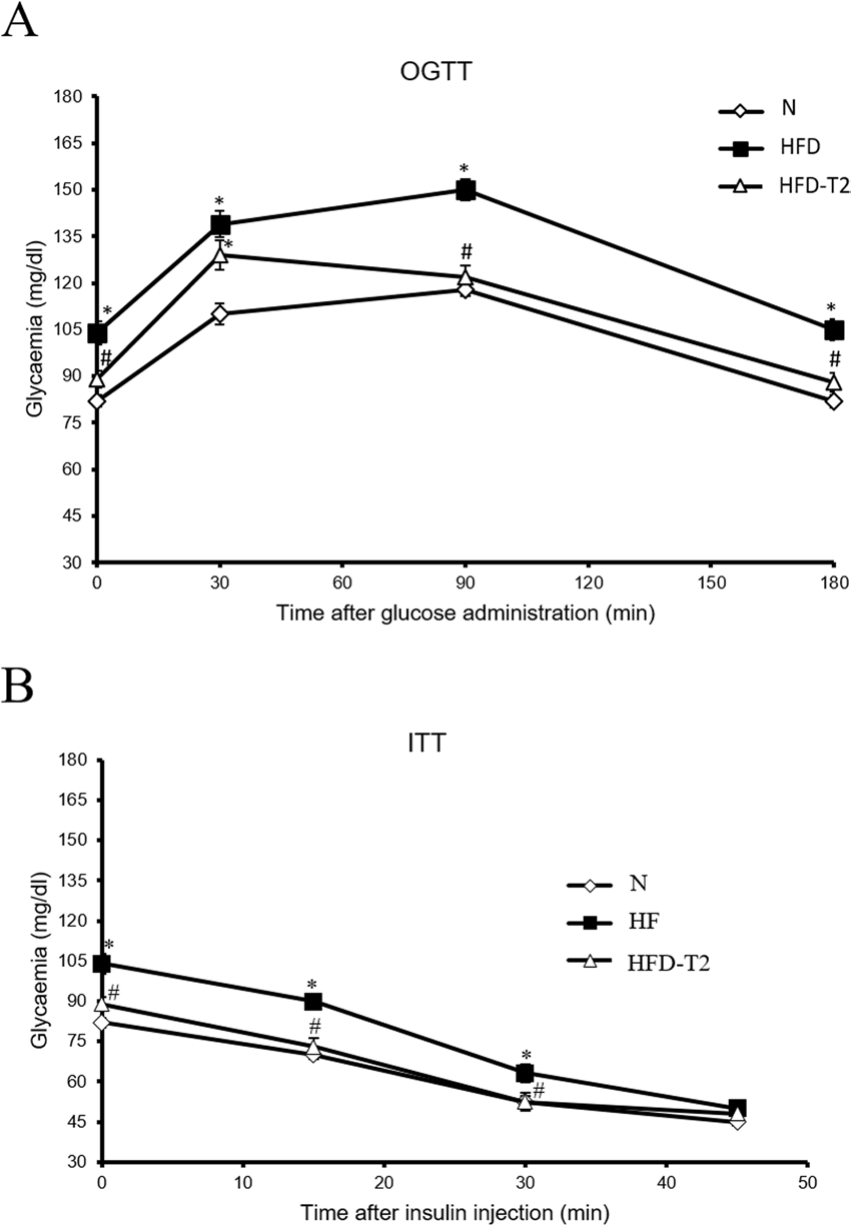
Glycaemia in N, HFD and HFD-T2 rats. (**A**) Oral Glucose Tolerance Test (OGTT) and (**B**) Insulin Tolerance Test (ITT) in rats fed on to standard diet (N), to high-fat diet (HFD) and to HFD with contemporary administration of T2 (HFD-T2). *p < 0.05 vs N; ^#^p < 0.05 vs HFD. Values are mean ± SEM of five independent treatments.

**Figure 2:**
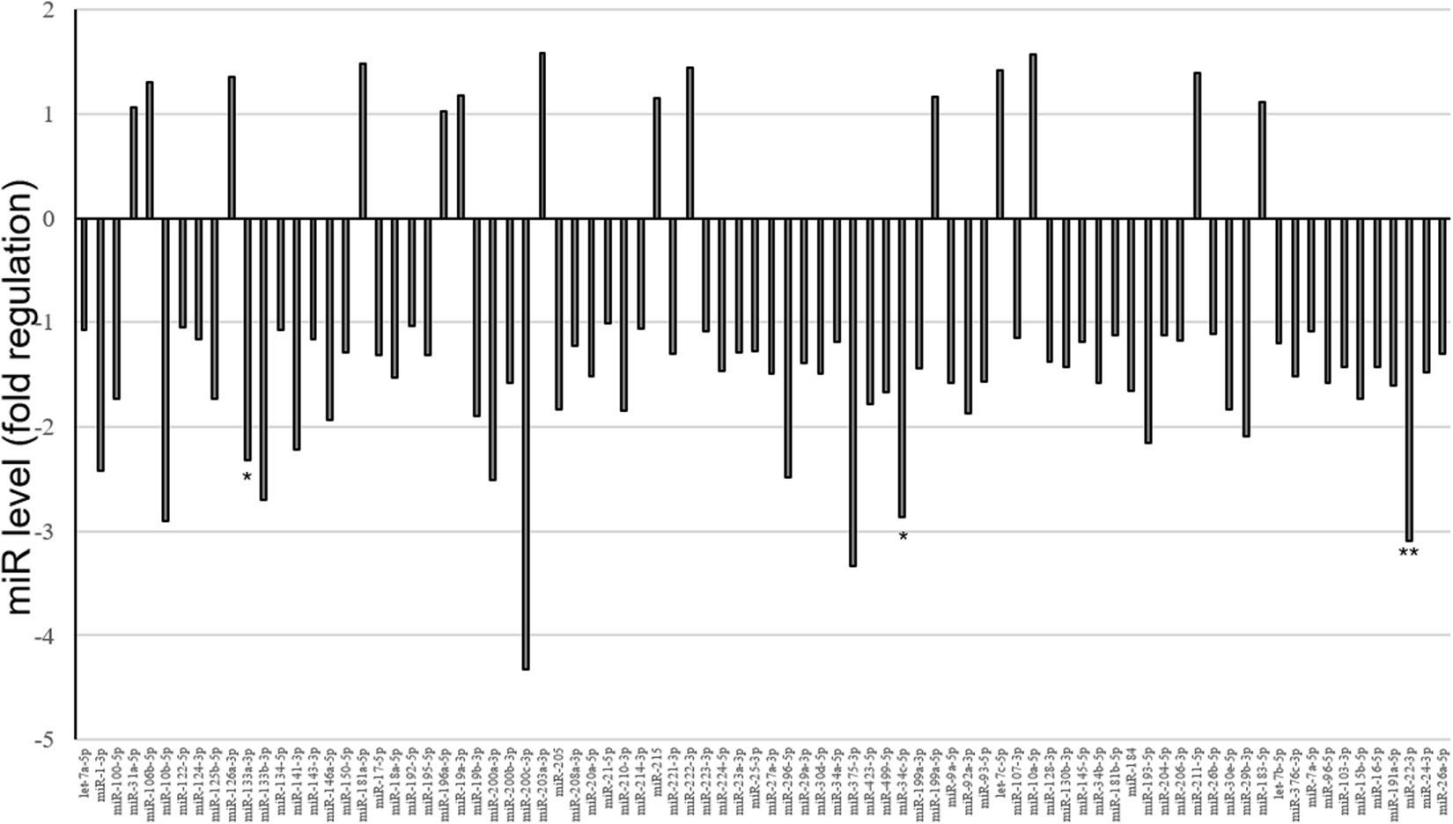
miRNA profiling. Fold regulation of miRNA levels detectable in the serum of 5 HFD-T2 treated rats versus 5 HFD rats (N = 5). P values at Student’s *t*-test were *p < 0.05, **p < 0.01.

HFD-T2 rats showed a glycaemia comparable to that of N animals (Fig. 1). In fact, the oral glucose tolerance test revealed that, after a glucose load, the animals treated with T2 had a better tolerance to this sugar compared to HFD rats (Fig. 1A). Furthermore, the insulin tolerance test showed that the reduction in the glycaemia after insulin administration was almost the same in HFD-T2 and N animals, but was impaired in HFD animals (Fig. 2B). These data indicate that T2 could prevent the development of insulin resistance induced by a hyperlipidic diet. Overall, the data indicate that T2 administration prevents body weight gain and increased adiposity without inducing a thyrotoxic state, and improve glucose tolerance and insulin sensitivity.

### Different circulating miRNAs were detected between HFD-T2 and HFD rats

**Figure 3:**
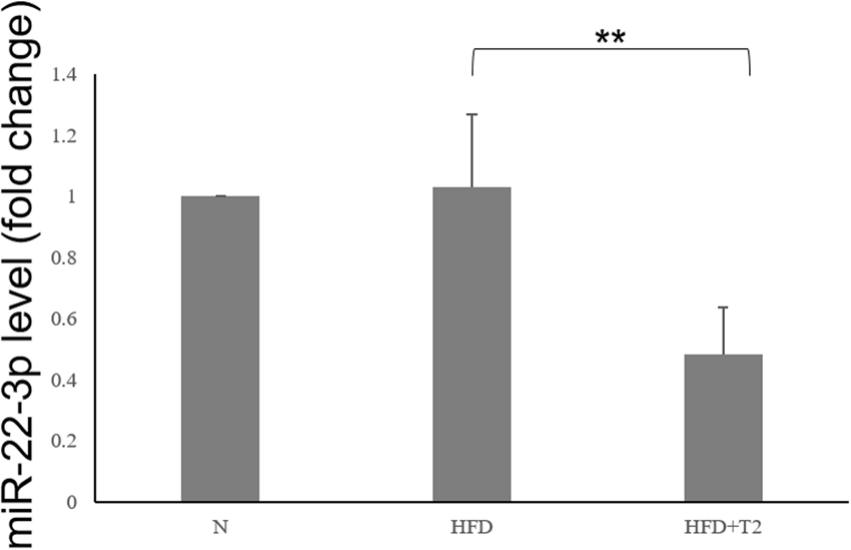
Circulating miR-22-3p. Serum samples were collected at 1 months from normally-fed rats (N), HFD-fed rats (HFD) and HFD-fed T2-treated rats. Relative miR-22 level was determined by RT-QPCR as described and reported as mean ± SEM of five independent experiments (N = 5) **p < 0.01.

In order to investigate whether the biological action of T2 was partly due to miRNA-based mechanisms, a screening of circulating miRNAs was performed on serum samples from HFD and from HFD-T2 animals, wherein the beneficial action of T2 was demonstrated. That screening revealed a decrease of several circulating miRNAs in the serum of HFD-T2 versus HFD animals, with miR-22-3p, miR-34c-5p, and miR-133a-3p significantly downregulated (3.1, 2.9 and 2.3, respectively) (Fig. 2). Then, the most responsive miRNA after T2 administration was further validated (Fig. 3). The data confirmed an approximately 50% decreased level of miR-22-3p (miR-22) in the serum of HFD-T2 rats in comparison to HFD rats, and similar level in the serum from HFD and N rats.

### *In vivo* T2-induced down-regulation of miR-22a-3p results in an up-regulation of Wnt-Responsive Transcription Factor Tcf7 with an impact on gluconeogenesis

**Figure 4:**
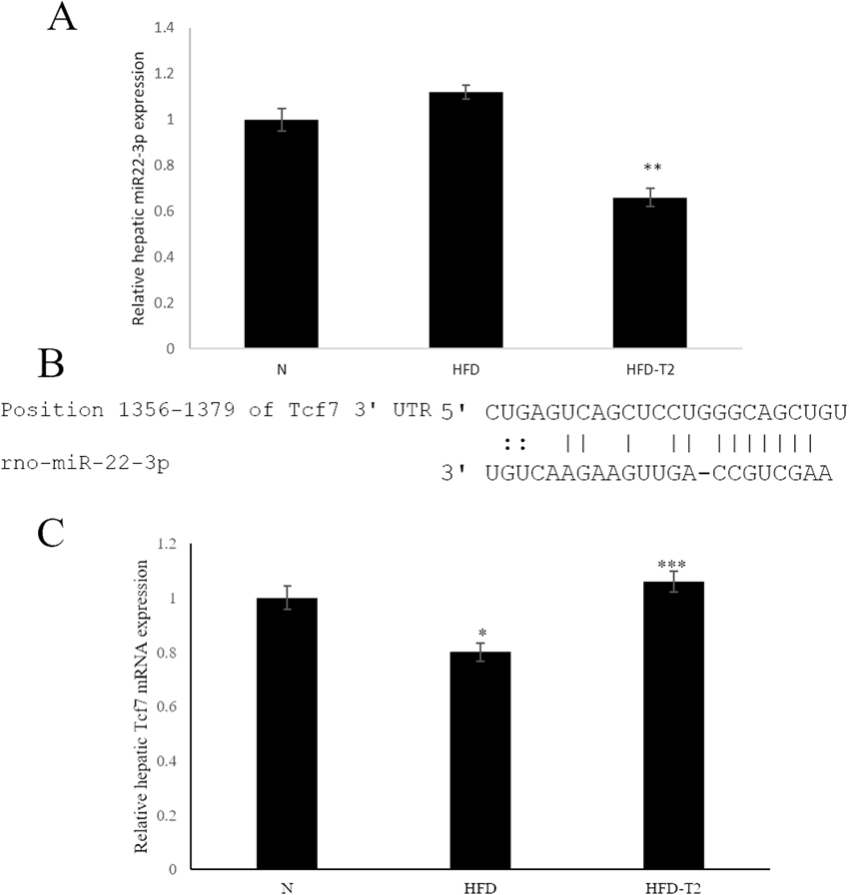
Hepatic expression of miR-22 and of its Tcf7 target. (**A**) RT-qPCR analysis of miR-22a in liver of N, HFD, HFD-T2 rats. (**B**) Predicted base-pairing between miR-22 and 3′UTR of Tcf7 mRNA. (**C**) Hepatic Tcf7 expression measured by RT-qPCR. Values are means ± SEM of five independent experiments (N = 5), *p < 0.05 vs N; ***P < 0.05 vs HFD; **P < 0.05 both vs N and HFD.

miR-22 is abundantly expressed in the liver^35,36^ and its expression was increased in animals models of insulin resistance and type 2 diabetes^37^. Moreover, miR-22 has been reported to have as direct target the Wnt-responsive transcription Factor Tcf7 in mouse and human cells, thus impairing gluconeogenesis and, as consequence, an increase in circulatory glucose level^37^. These data prompted us to investigate this pathway in our experimental rat model. First, miR-22 expression was measured in the liver, revealing that T_2_ administration to HFD-fed rats significantly reduced miR-22 expression when compared to HFD-fed rats; in HFD rats, the miR-22a expression was increased, although not significantly when compared to N animals (Fig. 4A). Then, based on the prediction of a conserved miR-22 binding site on rat Tcf7 3′UTR (Fig. 4B)^37^, the Tcf7 hepatic expression was measured, showing a reversed pattern with respect to that of miR-22, as expected for a target of the miRNA (Fig. 4C).

**Figure 5:**
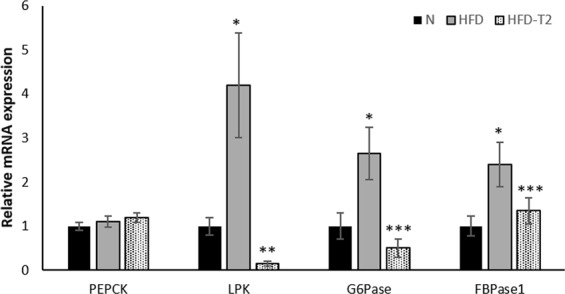
Hepatic expression of rate-limiting genes of gluconeogenesis. RT-qPCR analyses of glucose-6-phosphatase (G6Pase), fructose 1-6 bisphosphate (FBPase1) and phosphoenol pyruvate carboxykinase (*PEPCK)* were performed on RNA purified from the liver of N, HFD, HFD-T2 rats Values are means ± SEM of five independent experiments (N = 5),). **P < 0.05 vs N and HFD, *P < 0.05 vs N, ***P < 0.05 vs HFD.

Finally, in the same samples, we also analyzed the expression levels of genes of gluconeogenesis, i.e., glucose-6-phosphatase (G6Pase), fructose 1-6 bisphosphate (FBPase1) and phosphoenol pyruvate carboxykinase (*PEPCK)*, whose expression increased after Tcf7 targeting siRNA transfection of cell culture^37^. Consistently, in our *in vivo* model a strong decrease in the expression of G6Pase and FBPase was observed in response to T2 administration (Fig. 5), wherein Tcf7 resulted increased (Fig. 4C). Furthermore, T2 treatment prevented the increase in glycolytic enzyme (liver pyruvate kinase (LPK)) induced by hyperlipidic diet (Fig. 5).

## Discussion

It is well established that T2 exerts considerable effects on the metabolic activities. Indeed, several laboratories have demonstrated that it is able to influence both the energy metabolism and some biochemical pathways involved in lipid and glucose metabolism^9,10,13,15^. However, the mechanisms by which it carries out these actions must still be clarified. About glucose metabolism it is known that T2 showed beneficial effect on glucose tolerance and insulin-resistance^17,18^, as well as overweight/obesity and diabetes^16,17^. Recently, many reports have shown the involvement of aberrant miRNA expression in metabolic disorders. Because of this, in an attempt to reveal a possible link between miRNA regulation and the beneficial effects of T2 in an HFD-context, a screening of circulating miRNAs was performed on serum samples from HFD and HFD-T2 animals. In the present study for the first time, we provide evidences that miRNAs are involved in the actions exerted by T2 on glucose homeostasis.

The results revealed that the T2-treated animals are protected against fat accumulation and the resulting insulin resistance. In fact, the diiodothyronine prevented body weight gain and the increase in white adipose tissue mass induced by high fat diet; in addition, the diiodothyronine was able to avoid a serum lipid increase (see Table 1). By this way, T2 contributed to improve glucose tolerance and insulin sensitivity (see Fig. 1). As already shown by Lanni *et al*. using the same experimental design, these effects were not ascribed to central actions of T2 and its actions were not associated with thyrotoxicosis^16^. Regarding miRNAs, our data revealed a general decrease of miRNAs in the serum of HFD-T2 versus HFD animals, with miR-22-3p, miR-34c-5p, and miR-133a-3p significantly downregulated, suggesting, for the first time, that some biological actions of T2 could be at least in part due to miRNA-based mechanisms. Consistent with our data obtained with an *in vivo* model, some papers already linked the three miRNAs with glucose metabolism. In particular, miR-34c-5p was found abundantly expressed in the circulating monocytes of type 2 diabetes patients^38^. From the same miRNA family, miR-34a was down-regulated in type 2 diabetes patients after therapeutic resveratrol treatment^39^; again its expression was altered in obese db/db mice and its overexpression was associated with increased lipid accumulation in HepG2 cells^29^. With regard to miR-133a-3p, it was shown that diabetes leads to its downregulation, with an impact on the regulation of target key genes involved in cardiomyocyte hypertrophy, heart failure, cardiac arrhythmia and muscular dystrophy^40,41^. Finally, regarding to the most reduced miRNA in response to T2 treatment, miR-22 (-3.1-fold, HFD-T2 vs HFD), its increased hepatic expression has been reported in mouse models of insulin resistance and type 2 diabetes^25,42^, with Tcf7, a key transcription factor involved in the regulation of gluconeogenesis, being demonstrated as its target in mouse and human cells^31,43–45^. This pathway was further investigated in liver of our rat model, in the light of the effects of T2 in alleviating hyperglycemia, improving glucose tolerance and insulin sensitivity during HFD diet, a condition that impairs glucose homeostasis. We focalized our study principally on the liver because it is known that gluconeogenesis takes place mainly in the liver^46,47^, even if it cannot be ruled out that other insulin-resistant tissues could contribute to the level of circulating miR-22. The results showed that T2 administration to HFD-fed rats significantly reduced hepatic miR-22 expression when compared to HFD-fed rats alone. Then, based on the prediction of a conserved miR-22 binding site on rat Tcf7 3′UTR, the Tcf7 hepatic expression was measured, and accordingly with that reported for mouse and human cells^37^, revealed a reversed pattern with respect to that of miR-22, with a consequent reduced expression of Tcf7 targets (G6Pase and FBPase) that would result in a decrease of glucose release and improvement of glucose tolerance observed after T2 administration (Fig. 1). The reduction in miR-22 expression well correlates with the beneficial effects of T2 administration, since it has been found modulated in different metabolic disorders. Diniz GP *et al*. in 2017 showed that miR-22 is an important regulator of dyslipidemia as the loss of this miRNA attenuated the gain of fat mass and prevented dyslipidemia induced by hyperlipidic diet^48^. In addition, it has been shown the miR-22 involvement in cardiomyocyte hypertrophy and cardiac remodeling, probably by targeting SIRT1, a Nicotinamide Adenine Dinucleotide + -dependent histone deacetylase involved in a variety of biological processes, including energy metabolism, cell proliferation and chromatin dynamics^49,50^. Furthermore, we have already demonstrated that T2 prevented the increase in protein levels of LPK HFD-induced contributing to improve glucose tolerance^17^. Here we showed that T2 prevents that increase downregulating LPK mRNA levels suggesting that, in this way, the iodothyronine could maintain unaltered protein levels of LPK. Again, the effect on improvement of glucose metabolism could be, at least in part, due to an increase in resting metabolic rate as already reported by Lanni *et al*.^16^.

**Figure 6:**
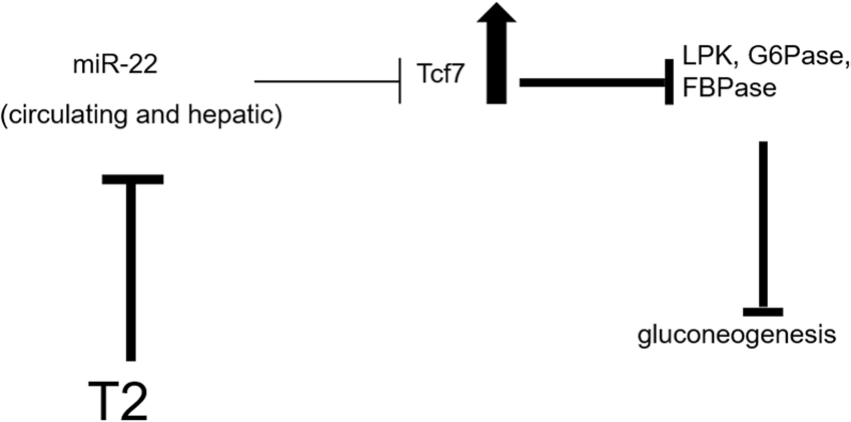
A model depicting the miR-22-mediated beneficial effect of T2 in HFD rats. The effect of T2 is represented by bold lines: the down-regulation of miR-22 in response to T2 treatment results in a reduced inhibitory effect on the expression of Tcf7 whose hepatic level increases, with the consequence of a reduced expression of gluconeogenesis enzymes and gluconeogenesis impairing.

In conclusion, our results suggest a miRNA-based action of T2, since different miRNAs changed their level in response to T2 administration; furthermore, our data indicate that one way through which T2 regulates glucose homeostasis could be the down-regulation of miR-22, resulting in an up-regulation of its Tcf7 target which, in turn, impairs gluconeogenesis by inhibiting the expression of gluconeogenesis enzymes (Fig. 6).

## Materials and Methods

### Animals and animal care

All animals received humane care according to the criteria outlined in the Guide for the Care and Use of Laboratory Animals prepared by the National Academy of Sciences and published by the National Institutes of Health. All animal protocols were approved by the Organism in charge of animal welfare (OBPA) of the University of Campania “Luigi Vanvitelli” (Caserta-Italy) and the Italian Minister of Health (Permit Number:704/2016-PR). Every effort was made to minimize animal pain and suffering. Male Wistar Rats (250–300 g, aged 8 weeks) were individually caged in a temperature-controlled room at 28 °C (thermoneutrality temperature for rats) under a 12-h light/12-h dark cycle. Before commencement of the study, a commercial mash (Charles River Laboratories, Calco, Italy) was available ad libitum and the animals had free access to water^17,51^. At the start of the study (day 0), after 7 days of acclimatizationto thermoneutrality, the rats were divided into three groups of 5 and treated for four weeks as follows:

- The first group (N) received a standard diet (total metabolizable percentage of energy: 60.4 carbohydrates, 29 proteins, 10.6 fat J J^−1^; 15.88 kJ gross energy g^−1^; Muscedola, Milan, Italy) with a daily injection intraperitoneally of vehicle^17,51^.
- The second group (HFD) received a high-fat diet (280 g diet supplemented with 395 g of lyophilized lamb meat [Liomellin, Milan, Italy], 120 g cellulose [Sigma-Aldrich, St. Louis, MO], 20 g mineral mix [ICN Biomedical, Solon, OH], 7 g vitamin mix [ICN], and 200 g low-salt butter [Lurpak, Denmark]; total metabolizable percentage of energy: 21 carbohydrates, 29 proteins, 50 fat J J^−1^; 19.85 kJ gross energy g^−1^) with a daily injection intraperitoneally of vehicle^17,51^.
- The third group (HFD-T2) received the same HFD with a daily injection of T2 (25 μg 100 g^−1^ body weight intraperitoneally) (Sigma-Aldrich)^17,51^.

All animals continued to have free access to water. At the end of the treatment, the rats were anesthetized by intraperitoneal injection of chloral hydrate (40 mg 100 g^−1^ body weight) and then decapitated. Liver, heart, gastrocnemius muscle, and abdominal white adipose tissue were excised, weighed, and immediately frozen in liquid nitrogen and stored at −80 °C for later processing^17,51^.

### Measurements of metabolic parameters

The serum levels of cholesterol and triglycerides were determined by standard procedures. For the oral glucose tolerance test (OGTT), rats were fasted overnight and then orally dosed with glucose (3 g/kg body wt) dissolved in water^17^. For the insulin tolerance test (ITT), rats were fasted for 5 h and then injected intraperitoneally with insulin (homolog rapid-acting, 10 units/kg body wt in sterile saline; Novartis, Basel, Switzerland)^17^. Samples of blood were collected before the oral glucose tolerance test and insulin tolerance test and at various times thereafter (as indicated in the Fig. 1), and glucose and insulin values were determined by means of a glucose monitor (BRIO, Ascensia, NY), calibrated for use with rats.

### RNA purification

200 μl of serum were used to extract total RNA, including small RNAs, by using miRNeasy Mini kit (Qiagen) according to the supplementary protocol provided for miRNA isolation from serum. A 5-μl aliquot of 5 nmol/l Syn-cel-miRNA-39 miScript miRNA Mimic was spiked into each sample before nucleic acid preparation to monitor the efficiency of miRNA recovery and to normalize miRNA expression in the subsequent real-time PCR analyses^52^.

Total RNA, including small RNAs, was also extracted by miRNeasy Mini kit (Qiagen) from liver tissues homogenized by polytron. The RNA was spectrophotometrically quantifed by NanoDrop 2000c, ThermoScientific.

### miRNA profiling

Reverse- transcription of RNA was performed by miScript II RT kit (SABiosciences); then, aliquots of cDNA preparation were used for real-time PCR profiling of mature miRNAs using Rat Serum&Plasma miRNA PCR Arrays (SABiosciences) in combination with miScript SYBR Green PCR kit (SABiosciences) and the MyiQ2 (Bio-Rad,Hercules, CA) instrument. Data analysis was performed with the web-based software package for the miRNA PCR array system (http://pcrdataanalysis.sabiosciences.com/mirna/arrayanalysis.php). In brief, Δ*C*t value for each miRNA profiled in a plate is calculated using the formula Δ*C*t = CtmiRNA− *C*tcel-miR-39. ΔΔCt for each miRNA across the two groups of samples (HFD and HFD T2 treated animals) is calculated using the formula: ΔΔ*C*t = Δ*C*t of HFD-T2 treated group − Δ*C*t of HFD group. Expression fold-change was then obtained as 2^−ΔΔ*C*t^ (the normalized gene expression (2^−Δ*C*t^) in the T2 treatment group divided the normalized gene expression (2^−ΔCt^) in the HFD group. Data are reported as fold-regulation, where fold-regulation is equal to the fold-change for fold-change values greater than one (up-regulation) or is the negative inverse of the fold-change for fold-change values lower than one (down-regulation)^52^. The replicate 2^−ΔCt^ values for each miRNA in the HFD and HFD-T2 treatment groups were statistically analyzed by Student’s t-test and the criteria of differential expression were p < 0.05.

### Real-time PCR analyses

miR-22 was detected and quantified by RT-QPCR with TaqMan® miRNA assays from Applied Biosystems according to the manufacturer’s protocol. Serum miR-22 level was normalized to that of *spiked-in* cel-miR-39 as described above; the expression level of hepatic miR-22 was normalized to that of RNU6B used as reference gene in the 2-ΔCt method^53^.

The hepatic expression levels of the other transcripts were determined by RT-QPCR. In particular, 1 μg of total RNA was used to generate cDNA strands in a 20-μl-reaction volume using the Super Script First Strand Synthesis System for RT-PCR (Invitrogen)^17^. An equivalent of 25 ng total cDNA was subsequently used in the amplification. Real-Time quantitative RT-PCR (QRT-PCR) was carried out with 50 nM gene-specific primers and IQ SYBR Green supermix (Biorad) using standard cycle parameters on a MyiQ2 (BioRad). A melting curve analysis was completed following amplification from 55–95 °C to assure product identification and homogeneity. Each sample was repeated in triplicate and was normalized to the housekeeping gene β-actin to compensate for any differences in starting quantity of total RNA.

PCR primers were designed by using the Primer 3 program (Untergasser *et al*., 2012), and synthesized and verified by sequencing at Eurofins Genomics (Ebersberg, Germany)^51^. Primers used were the following:

**rβ-ACT sense** 5′-GGA GAT TAC TGC CCT GGC TCC TA-3′

**rβ-ACT Antisense** 5′-GAC TCA TCG TAC TCC TGC TTG CTG-3′

**rG6Pase sense** 5′-CAG TGG TTG GAG ACT GGT TC3′

**rG6Pase antosense** 5′ TTT CCA CGA AAG ATA GCG AGA 3′

**rLPK sense** 5′-GCA TTG AAA GTG GAA AGC TC-3′

**rLPK antisense** 5′-GGG GCT AGA TGG CAG ATG TA-3′

**rPEPCK sense** 5′-CTG GCG TTG AAT GCT TTC TC-3′

**rPEPCK antisense** 5′-CAA CTG TTG GCT GGC TCT CA-3′

**rTCF7 sense**5′-AGG TCA GAT GGG TTG GAC TG-3′

**rTCF7 antisense**5′-AGG GTG CAC ACT GGG TTT AG-3′

**rFBPase sense** 5′-ATGGTGGAC-CATGCACCCTTCGAA-3′

**rFBPase antisense** 5′-TTTCTAATT-CCCCGTCGTGGCAAGTCC-3′

### Statistical analysis

Results are expressed as mean ± SEM. The statistical significance of differences between N, HFD and HFD-T2 groups (n = 5) was determined using one-way ANOVA followed by a Student-Newman-Keuls test^51^. Differences were considered significant at p < 0.05. The statistical significance of differences between HFD and HFD-T2 groups (n = 5) was determined using Student’s *t*-test. Differences were considered significant at p < 0.05.

### Statement of ethics

Animal experiments conform to internationally accepted standards. All animals received humane care according to the criteria outlined in the Guide for the Care and Use of Laboratory Animals prepared by the National Academy of Sciences and published by the National Institutes of Health. All animal protocols were approved by the Organism in Charge of Animal Welfare (OBPA) of the University of Campania “Luigi Vanvitelli” (Caserta-Italy) and the Italian Minister of Health (Permit Number:704/2016-PR).

## References

[1] Mullur R, Liu YY, Brent GA (2014). Thyroid hormone regulation of metabolism. Physiol Rev.

[2] Vaitkus JA, Farrar JS, Celi FS (2015). Thyroid Hormone Mediated Modulation of Energy Expenditure. Int J Mol Sci.

[3] Moreno M (2008). Metabolic effects of thyroid hormone derivatives. Thyroid.

[4] Senese R, Cioffi F, de Lange P, Goglia F, Lanni A (2014). Thyroid: biological actions of ‘nonclassical’ thyroid hormones. J Endocrinol.

[5] Senese R, Lasala P, Leanza C, de Lange P (2014). New avenues for regulation of lipid metabolism by thyroid hormones and analogs. Front Physiol.

[6] Zucchi R, Accorroni A, Chiellini G (2014). Update on 3-iodothyronamine and its neurological and metabolic actions. Front Physiol.

[7] Goglia F (2014). The effects of 3,5-diiodothyronine on energy balance. Front Physiol.

[8] Davis PJ, Goglia F, Leonard JL (2016). Nongenomic actions of thyroid hormone. Nat Rev Endocrinol.

[9] Cavallo A (2016). Acute administration of 3,5-diiodo-L-thyronine to hypothyroid rats stimulates bioenergetic parameters in liver mitochondria. J Bioenerg Biomembr.

[10] Iannucci LF (2017). Metabolomic analysis shows differential hepatic effects of T2 and T3 in rats after short-term feeding with high fat diet. Sci Rep.

[11] Sacripanti G (2018). 3,5-Diiodo-l-Thyronine Increases Glucose Consumption in Cardiomyoblasts Without Affecting the Contractile Performance in Rat Heart. Front Endocrinol (Lausanne).

[12] Cioffi F, Senese R, Lanni A, Goglia F (2013). Thyroid hormones and mitochondria: with a brief look at derivatives and analogues. Mol Cell Endocrinol.

[13] Lanni A, Moreno M, Goglia F (2016). Mitochondrial Actions of Thyroid Hormone. Compr Physiol.

[14] Rutigliano G, Zucchi R (2017). Cardiac actions of thyroid hormone metabolites. Mol Cell Endocrinol.

[15] Louzada RA, Carvalho DP (2018). Similarities and Differences in the Peripheral Actions of Thyroid Hormones and Their Metabolites. Front Endocrinol (Lausanne).

[16] Lanni A (2005). 3,5-diiodo-L-thyronine powerfully reduces adiposity in rats by increasing the burning of fats. FASEB J.

[17] de Lange P (2011). Nonthyrotoxic prevention of diet-induced insulin resistance by 3,5-diiodo-L-thyronine in rats. Diabetes.

[18] Moreno M (2011). 3,5-Diiodo-L-thyronine prevents high-fat-diet-induced insulin resistance in rat skeletal muscle through metabolic and structural adaptations. FASEB J.

[19] Pietzner M (2015). Urine Metabolomics by (1)H-NMR Spectroscopy Indicates Associations between Serum 3,5-T2 Concentrations and Intermediary Metabolism in Euthyroid Humans. Eur Thyroid J.

[20] Pietzner M (2015). Translating pharmacological findings from hypothyroid rodents to euthyroid humans: is there a functional role of endogenous 3,5-T2?. Thyroid.

[21] Hammond SM (2015). An overview of microRNAs. Adv Drug Deliv Rev.

[22] Potenza N, Russo A (2013). Biogenesis, evolution and functional targets of microRNA-125a. Mol Genet Genomics.

[23] Coppola N (2017). Lowered expression of microRNA-125a-5p in human hepatocellular carcinoma and up-regulation of its oncogenic targets sirtuin-7, matrix metalloproteinase-11, and c-Raf. Oncotarget.

[24] Soifer HS, Rossi JJ, Saetrom P (2007). MicroRNAs in disease and potential therapeutic applications. Mol Ther.

[25] Kornfeld JW (2013). Obesity-induced overexpression of miR-802 impairs glucose metabolism through silencing of Hnf1b. Nature.

[26] Defronzo R. A. (2004). Dysfunctional fat cells, lipotoxicity and type 2 diabetes. International Journal of Clinical Practice.

[27] DeFronzo Ralph A (2004). Pathogenesis of type 2 diabetes mellitus. Medical Clinics of North America.

[28] Mirra P (2018). The Destiny of Glucose from a MicroRNA Perspective. Front Endocrinol (Lausanne).

[29] Kaur K, Pandey AK, Srivastava S, Srivastava AK, Datta M (2011). Comprehensive miRNome and in silico analyses identify the Wnt signaling pathway to be altered in the diabetic liver. Mol Biosyst.

[30] Behari J (2010). The Wnt/beta-catenin signaling pathway in liver biology and disease. Expert Rev Gastroenterol Hepatol.

[31] Liu H (2011). Wnt signaling regulates hepatic metabolism. Sci Signal.

[32] Taniguchi CM, Emanuelli B, Kahn CR (2006). Critical nodes in signalling pathways: insights into insulin action. Nat Rev Mol Cell Biol.

[33] Silvestri E (2005). Thyroid-hormone effects on putative biochemical pathways involved in UCP3 activation in rat skeletal muscle mitochondria. FEBS Lett.

[34] Mollica MP (2009). 3,5-diiodo-l-thyronine, by modulating mitochondrial functions, reverses hepatic fat accumulation in rats fed a high-fat diet. J Hepatol.

[35] Jordan SD (2011). Obesity-induced overexpression of miRNA-143 inhibits insulin-stimulated AKT activation and impairs glucose metabolism. Nat Cell Biol.

[36] Landgraf P (2007). A mammalian microRNA expression atlas based on small RNA library sequencing. Cell.

[37] Kaur K (2015). Elevated Hepatic miR-22-3p Expression Impairs Gluconeogenesis by Silencing the Wnt-Responsive Transcription Factor Tcf7. Diabetes.

[38] Baldeon RL (2015). Type 2 Diabetes Monocyte MicroRNA and mRNA Expression: Dyslipidemia Associates with Increased Differentiation-Related Genes but Not Inflammatory Activation. PLoS One.

[39] Tome-Carneiro J (2013). One-year supplementation with a grape extract containing resveratrol modulates inflammatory-related microRNAs and cytokines expression in peripheral blood mononuclear cells of type 2 diabetes and hypertensive patients with coronary artery disease. Pharmacol Res.

[40] Yu H, Lu Y, Li Z, Wang Q (2014). microRNA-133: expression, function and therapeutic potential in muscle diseases and cancer. Curr Drug Targets.

[41] Feng B, Chen S, George B, Feng Q, Chakrabarti S (2010). miR133a regulates cardiomyocyte hypertrophy in diabetes. Diabetes Metab Res Rev.

[42] Trajkovski M (2011). MicroRNAs 103 and 107 regulate insulin sensitivity. Nature.

[43] Noble JA (2003). A polymorphism in the TCF7 gene, C883A, is associated with type 1 diabetes. Diabetes.

[44] Erlich HA (2009). Evidence for association of the TCF7 locus with type I diabetes. Genes Immun.

[45] Nakae J, Park BC, Accili D (1999). Insulin stimulates phosphorylation of the forkhead transcription factor FKHR on serine 253 through a Wortmannin-sensitive pathway. J Biol Chem.

[46] Edgerton DS, Johnson KM, Cherrington AD (2009). Current strategies for the inhibition of hepatic glucose production in type 2 diabetes. Front Biosci (Landmark Ed).

[47] Wahren J, Ekberg K (2007). Splanchnic regulation of glucose production. Annu Rev Nutr.

[48] Diniz GP (2017). Loss of microRNA-22 prevents high-fat diet induced dyslipidemia and increases energy expenditure without affecting cardiac hypertrophy. Clin Sci (Lond).

[49] Fiore D (2016). PDE5 Inhibition Ameliorates Visceral Adiposity Targeting the miR-22/SIRT1 Pathway: Evidence From the CECSID Trial. J Clin Endocrinol Metab.

[50] Huang ZP (2013). MicroRNA-22 regulates cardiac hypertrophy and remodeling in response to stress. Circ Res.

[51] Senese R (2017). Both 3,5-Diiodo-L-Thyronine and 3,5,3′-Triiodo-L-Thyronine Prevent Short-term Hepatic Lipid Accumulation via Distinct Mechanisms in Rats Being Fed a High-Fat Diet. Front Physiol.

[52] Stiuso P (2015). MicroRNA-423-5p Promotes Autophagy in Cancer Cells and Is Increased in Serum From Hepatocarcinoma Patients Treated With Sorafenib. Mol Ther Nucleic Acids.

[53] Potenza N (2017). MicroRNA-125a-5p Is a Downstream Effector of Sorafenib in Its Antiproliferative Activity Toward Human Hepatocellular Carcinoma Cells. J Cell Physiol.

